# Computer Vision-Based Deep Learning Modeling for Salmon Part Segmentation and Defect Identification

**DOI:** 10.3390/foods14203529

**Published:** 2025-10-16

**Authors:** Chunxu Zhang, Yuanshan Zhao, Wude Yang, Liuqian Gao, Wenyu Zhang, Yang Liu, Xu Zhang, Huihui Wang

**Affiliations:** 1 College of Biosystems Engineering and Food Science, Zhejiang University, Hangzhou 310027, China; 747189502zcx@gmail.com; 2 School of Mechanical Engineering & Automation, Dalian Polytechnic University, Dalian 116039, China; zhaoyuanshan2024@163.com (Y.Z.); 18741357362@163.com (W.Y.); glqjy9@163.com (L.G.); zwy20241029@163.com (W.Z.); ly.19880126@163.com (Y.L.)

**Keywords:** salmon, part segmentation, defect detection, two-stage model, machine vision

## Abstract

Accurate cutting of salmon parts and surface defect detection are the key steps to enhance the added value of its processing. At present, mainstream manual inspection methods have low accuracy and efficiency, making it difficult to meet the demands of industrialized production. A machine vision inspection method based on a two-stage fusion network is proposed in this paper, aiming to achieve accurate cutting of salmon parts and efficient recognition of defects. The fish body image is collected by building a visual inspection system, and the dataset is constructed by preprocessing and data enhancement. For the part cutting, the improved U-Net model that introduces the CBAM attention mechanism is used to strengthen the extraction ability of the fish body texture features. For defect detection, the two-stage fusion architecture is designed to quickly locate the defective region by adding the YOLOv5 of the P2 small target detection layer first, and then the cropped region is fed into the improved U-Net for accurate cutting. The experimental results demonstrate that the improved U-Net achieves a mean average precision (mAP) of 96.87% and a mean intersection over union (mIoU) of 94.33% in part cutting, representing improvements of 2.44% and 1.06%, respectively, over the base model. In defect detection, the fusion model attains an mAP of 94.28% with a processing speed of 7.30 fps, outperforming the single U-Net by 28.02% in accuracy and 236.4% in efficiency. This method provides a high-precision, high-efficiency solution for intelligent salmon processing, offering significant value for advancing automation in the aquatic product processing industry.

## 1. Introduction

Salmon is popular globally as an aquatic product of great economic and nutritional value. In 2022, the global salmon market size continued to grow and is expected to surpass USD 30 billion by 2027, at a Compound Annual Growth Rate (CAGR) of over 8% [1]. Fine segmentation of salmon is the core link to enhance the value of raw materials, which mainly includes key processes such as part segmentation and defect detection [2]. Part segmentation precisely divides salmon into market-driven portions (e.g., belly, loin, back, and tail) to accommodate diverse culinary requirements, enhance product value, and minimize waste. Concurrently, defect detection identifies and removes surface imperfections (such as bruises or fat deposits), thereby improving sensory quality (taste and appearance) and ensuring consistent product standards [3]. However, existing methods for part segmentation and defect detection still face critical limitations in accuracy, efficiency, and adaptability to biological variability [4].

In current industrial practice, part segmentation remains heavily dependent on manual intervention. Operators typically identify anatomical regions by visually assessing texture and other morphological features, which would lead to inconsistent cutting results and even increased scrap material [5]. Despite the availability of advanced mechanized systems (such as the 184 header and Portion Cut series) in the international market, their capabilities remain predominantly limited to gross, geometry-based processing, including fin trimming, heading, and quantitative sectioning based on fixed lengths or weights. These systems lack the integrated computer vision and intelligence necessary for fine part division based on biological texture features, resulting in significant deviation of the division boundary [6]. Defects such as parasites, cuts, and color changes are relatively obvious and are directly identified and removed during fillet process. For defects like blood spots and fat, due to their large quantity, they are typically identified at specialized workstations. However, these defects are characterized by their minute size, random distribution, and visual similarity to normal tissue, leading to persistently high rates of missed detection. Tiny defects are virtually impossible to detect [7]. In the part segmentation and surface trimming sessions, machine vision combined with traditional machine learning methods has been applied, but these approaches have significant limitations [8]. Due to complex texture and individual variations in salmon, traditional machine learning struggles to capture subtle differences between parts (e.g., belly, loin, back). This leads to significant segmentation boundary errors, poor batch consistency, and low raw material utilization. Especially for the defect detection, existing methods extract defect features through traditional algorithms such as principal component analysis (PCA) and linear discriminant analysis (LDA), and then locate the defects through the classification model, but due to the weak feature differences between the defects and the normal texture, the traditional feature extraction capability is limited, resulting in sparse feature points, insufficient fit between the predicted contours and the real defect edges, large boundary errors, and a high rate of leakage detection, which not only affect the accurate removal of defective areas by the cutting equipment, but also reduce the appearance quality of the product due to residual defects [9]. Therefore, achieving accurate part segmentation and efficient defect detection represents a critical challenge for the salmon processing industry, as it is essential for ensuring processing consistency and maximizing raw material utilization [10].

The inherent biological diversity of fish and other meat products results in complex and variable image characteristics. Deep learning is particularly well-suited for these biological contexts because it can automatically learn hierarchical and abstract features directly from image data. This capability provides distinct advantages in addressing intricate nonlinear texture patterns and natural biological variations, which are common in tasks such as salmon portion segmentation and defect detection [11]. In the context of the rapid development of computer vision technology, the research on the application of deep learning models in the field of fish processing has made significant progress. In particular, image segmentation models and target detection algorithms based on the encoder–decoder structure provide a new technological path for fish processing automation due to their excellent feature extraction capabilities. Deep learning has demonstrated significant potential in tasks directly analogous to food quality control, such as fine part segmentation and defect identification [12]. For instance, in the context of food part segmentation, a study by Zhang et al. (2025) utilized a U-Net architecture to precisely segment chicken breasts into distinct muscle sections based on tissue texture, demonstrating the model’s capability for fine-grained anatomical partitioning in food products [13]. Similarly, for defect detection, a study by Wang et al. (2025) utilized an improved YOLOv5 model to identify and localize surface bruising on apples, demonstrating the model’s efficacy in detecting subtle defects against variable backgrounds [14]. These examples underscore the suitability of deep learning frameworks for the challenges of salmon part segmentation and defect detection, as they effectively handle the intricacies of biological variability and subtle feature differentiation. U-Net, as a deep learning model widely used in image segmentation, has excellent performance in the field of image segmentation, especially in the prediction accuracy [15], but the basic U-Net framework has insufficient ability to recognize tiny targets and limited generalization ability, which makes it difficult to accurately deal with the tiny and randomly distributed defects on the surface of the salmon, resulting in insufficient segmentation contour fit [16]; and YOLOv5, as a single-stage target detection model, YOLOv5 can quickly localize the target and meet the real-time demand, but it cannot provide pixel-level fine contours. This limitation makes it difficult to meet high-precision segmentation requirements [17]. Therefore, given the unique advantages of these two types of models, a potential two-stage approach could be developed, in which YOLOv5 is used to quickly localize the defective region to reduce redundant calculations, and then the localized region is inputted into the improved U-Net for accurate segmentation. It may take both efficiency and accuracy into account and meet the requirements of fine cutting of salmon.

**Figure 1:**
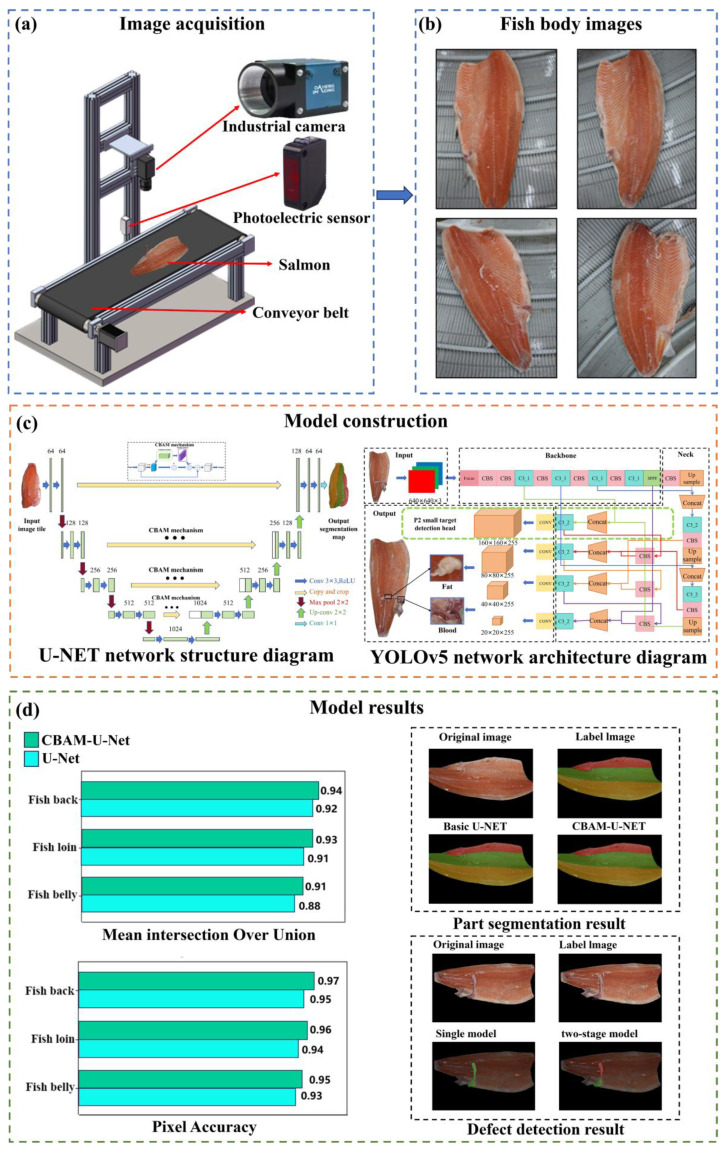
Main flow of the research: (**a**) image acquisition; (**b**) fish body image; (**c**) model construction; (**d**) model results.

This study addresses the challenges of inaccurate part segmentation and inefficient defect detection in salmon processing by developing a deep learning and machine vision model that utilizes shape, texture, and color features, thereby enabling precise segmentation and providing a theoretical and technical foundation for automation. To achieve these objectives, this study proposes the following methodological contributions: First, a visual inspection system was built to collect images of preliminarily dissected salmon bodies, leading to the construction of dedicated part segmentation and defect detection datasets that provide crucial data support for algorithm development. Second, for precise part identification, the U-Net architecture was optimized by integrating the Convolutional Block Attention Module (CBAM), which enhances feature responses in critical areas such as fish body edges and textural protrusions. Third, a novel two-stage detection model was constructed by combining YOLOv5, modified with a P2 (a high-resolution feature layer dedicated to small object detection) layer for small object detection, with the CBAM-enhanced U-Net; this framework first rapidly locates potential defect regions and then performs their precise contour segmentation, with its effectiveness confirmed through comparative analysis against baseline model. The main flow of this paper is shown in Figure 1.

## 2. Materials and Methods

### 2.1. Sample Preparation

The salmon samples required for the experiment were obtained from the production line of a certain aquatic product processing company in Shanghai, as shown in Figure 1a. The image collection was conducted over three distinct batches of fish raw materials to incorporate natural variability in raw materials. The salmon supplied to this facility originated from multiple sources (including farms in Norway and Chile), ensuring diversity in breed and growth conditions. Collection system primarily consisted of an industrial camera (MER-503-20U3C, Daheng Imaging, Beijing, China) with a resolution of 2448 × 2048 pixels, equipped with a 16 mm focal length lens. Image capture was triggered by an E3Z-T81 single-beam infrared photoelectric sensor, which detected the presence of a fish body on the conveyor belt. After pretreatment of filleting and skinning, two fish bodies (Figure 1b) triggered the photoelectric switch on the conveyor belt, and the camera captured the images. The length of the fish fillets ranged from 60 to 70 cm. Following common practice in the field and considering the application of extensive data augmentation [18], a total of 1529 salmon body images were obtained. The images are all from different salmon samples. To simplify the data acquisition system and adapt to complex machining environments, all images were captured under the existing ambient lighting conditions of the machining workshop. Subsequent image processing and enhancement techniques were employed to mitigate the impact of varying lighting conditions on model robustness. The above images are pre-processed in Section 2.2 and converted to PNG format with 512 × 512 pixels, which are used as the input for modeling the part recognition and segmentation of fish bodies.

### 2.2. Image Preprocessing

**Figure 2:**
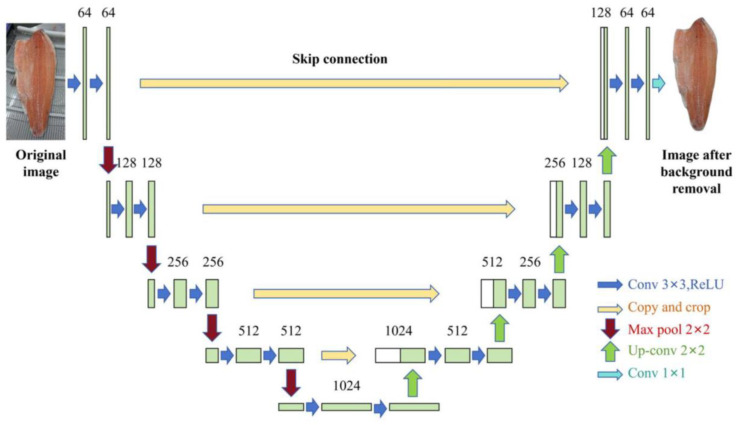
Base U-Net network structure.

To improve the overall quality of the dataset and ensure data consistency, image preprocessing is required, including background removal and adjustments to brightness and contrast. The original images are uniformly resized to 512 × 512 pixels using bicubic interpolation to ensure consistency in input data specifications [19]. Background removal was performed using a deep learning segmentation model based on basic U-Net architecture, which was end-to-end trained for this task on the dataset of 1529 images with pixel-accurate ground truth. The model was trained using the Adam optimizer with a learning rate of 0.001 and a batch size of 16 [20]. As shown in Figure 2, the network structure is based on the “U-shaped” encoder–decoder structure as the core, can effectively capture the texture differences on the surface of the salmon, such as the texture demarcation line between the belly of the fish and the loin of the fish, through the step-by-step extraction and restoration of features, providing a fine feature support for the segmentation of the parts of the fish [21]. The encoder is located on the left side of the U-shaped structure in Figure 2, and is responsible for extracting hierarchical features from the input image, which generates a 64-channel feature map after an initial 3 × 3 convolution + ReLU operation, followed by downsampling through 2 × 2 maximal pooling, where the feature map size is halved for every downsampling and the number of channels is doubled, and a 1024-channel high-level semantic feature map is finally formed at the end of the encoder [22]. In this process, the model gradually focuses on the global features of the fish body to provide basic features for distinguishing the macroscopic regions such as the dorsal and ventral regions of the fish. The decoder is located on the right side of the U-shaped structure in Figure 2, and assumes the function of feature reduction and fine segmentation, which is achieved by up-sampling through 2 × 2 inverse convolution, doubling the size of the feature map and halving the number of channels at each up-sampling step [23]. At the same time, with the help of the “Copy and crop” jump connection labeled in Figure 2, the feature maps sampled from the decoder are spliced and fused with the feature maps of the same resolution in the encoder, which integrates the underlying details retained by the encoder, such as the fish texture bumps, and gradually restores the spatial information of the image after 3 × 3 convolution + ReLU optimization, so that the segmentation results are more closely matched to the actual boundary. After that, the image spatial information is gradually recovered, so that the segmentation results are closer to the actual boundary [24]. In the output layer, the final feature map generated by the decoder is mapped to the segmentation result of the same size as the input image by 1 × 1 convolution, which realizes the accurate classification of the body and background of the fish. For reflective and dark areas on the fish body surface, gamma correction is used to adjust brightness, and contrast-constrained adaptive histogram equalization (CLAHE) technology is applied to enhance texture details, thereby improving texture clarity. To enhance the model’s robustness and compensate for the limited dataset size, aggressive online data augmentation was applied during the training phase. This included random variations in image brightness (±20%), contrast (±20%), hue (±10%), and saturation (±20%) to simulate different lighting and appearance conditions. Geometric augmentations such as random rotation (±15 degrees), scaling (80–120%), and horizontal flipping were also used to improve invariance to object pose and size, thereby enhancing model generalization capabilities and providing high-quality data support for subsequent network models [25].

### 2.3. Data Set Construction

Four datasets were established and used for image background removal and part identification segmentation based on U-Net, defect detection based on YOLOv5 and the two-stage detection modeling, respectively. All images were classified and labeled by Labelme (version 5.4.1) and LabelImg (version 1.8.6) labeling software, and the part segmentation dataset required manual contouring of the belly, loin, and back of the fish body. The defect detection dataset is labeled by two types of defects: fat and bruise [26]. A total of 1529 salmon body images were collected and used to construct multiple datasets for different tasks. Background segmentation was performed on all images (1529) using U-Net. From these, a dataset of 489 images was built for body part segmentation with U-Net, and another set of 424 images was prepared for defect detection using YOLOv5. A combined dataset of 616 images was also established for defect detection and segmentation using a two-stage U-Net and YOLOv5 framework. To ensure the independence of the splits, all datasets were divided into training, validation, and test sets at the unique fish specimen level following an 8:1:1 ratio. The specific purpose, size, and composition of each dataset are summarized in Table 1.

### 2.4. Part Identification Segmentation Model Based on Improved U-Net

**Figure 3:**
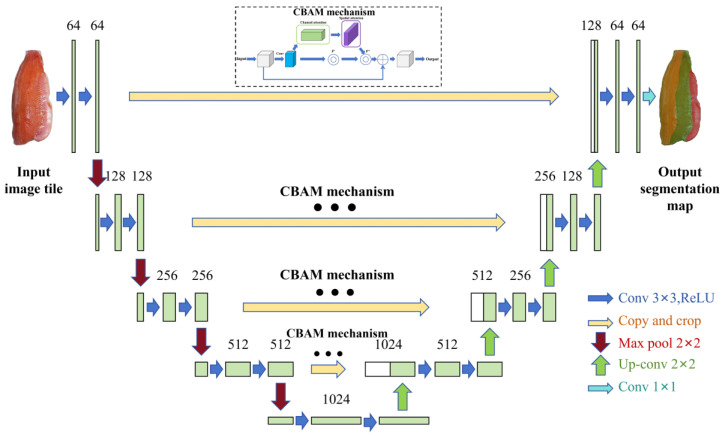
Network structure with CBAM mechanism added.

**Figure 4:**
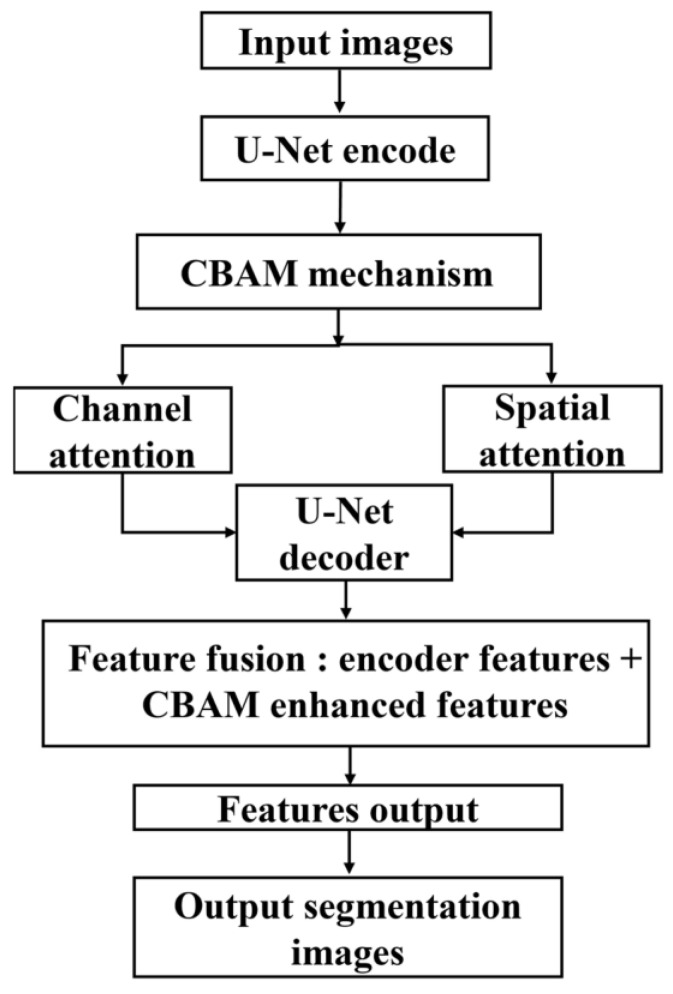
CBAM attention mechanism workflow.

The part identification segmentation model was established to identify and segment parts such as the fish belly, loin, and back, thereby providing precise coordinates for subsequent tool part cutting. The model continues to use the basic framework structure of U-Net as described in Section 2.2. In order to further improve the performance of the model in the tasks of site segmentation and defect detection, the CBAM (Convolutional Block Attention Module) attention mechanism was integrated into the feature extraction network of U-Net, which enables the model to select and evaluate the texture and color features of the salmon body in a more detailed way, thus enhancing its target region perception of the target area. CBAM is a lightweight attention mechanism that sequentially generates attention maps for both channel and spatial dimensions. It amplifies informative features through a channel attention module that adaptively recalibrates channels (‘what’) and a spatial attention module that locates key regions (‘where’). This dual-path structure enables the model to focus on the most critical features of complex regions and minute defects. As shown in Figure 3, the network structure with the added attention mechanism is mainly composed of two modules: Channel Attention and Spatial Attention. The channel attention module enhances the representation of important feature channels by adaptively adjusting the weights of each channel, while the spatial attention module enhances the feature response of key regions by evaluating the importance of each location in the image. It has been shown that the attentional mechanism can improve the efficiency and accuracy of the model in processing visual information by weighting low-level information [27]. As shown in Figure 4, The workflow of the CBAM attention mechanism is illustrated in the figure, which provides a structured overview of its operation.

### 2.5. Two-Stage Defect Detection Model

#### 2.5.1. Two-Stage Defect Detection Model Architecture

**Figure 5:**
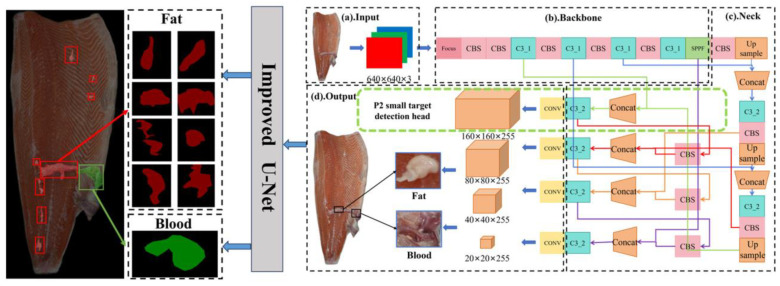
Two-stage detection model architecture diagram.

**Figure 6:**
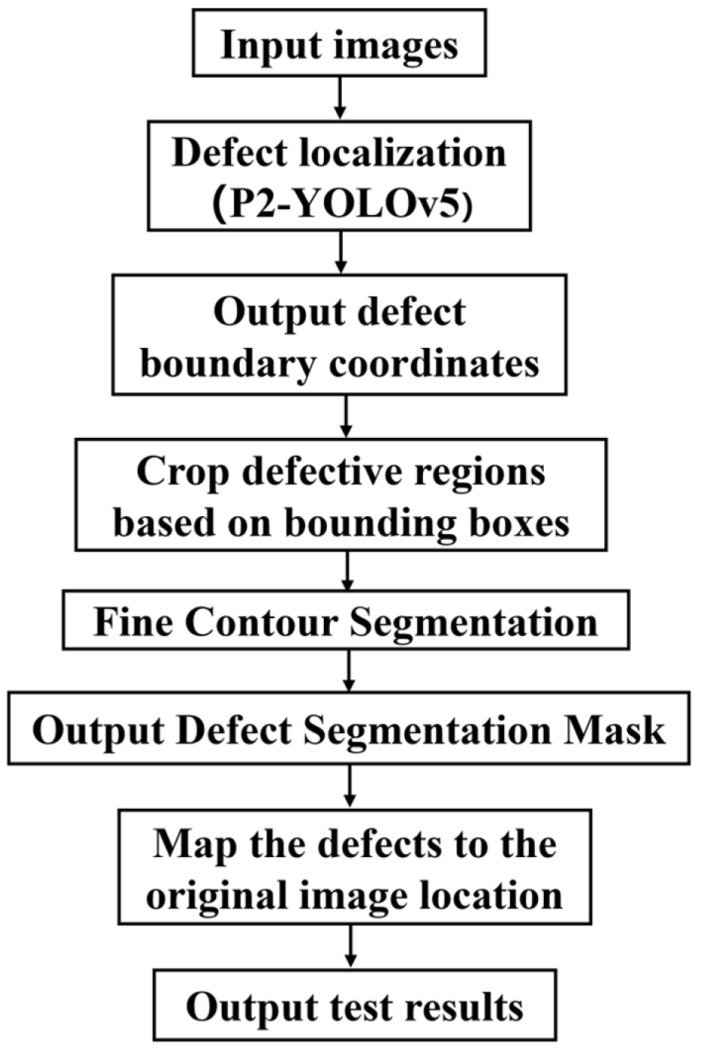
Two-stage detection model workflow.

YOLOv5 shows significant advantages in target detection tasks due to its efficient prediction speed and high detection accuracy. However, this type of model can only realize the initial localization of defects, and cannot carry out more accurate prediction of the segmentation contour. This limitation makes it difficult to meet the requirements for high-precision defect cutting. In contrast, U-Net, as a classical image segmentation network, is capable of high-precision defect boundary delineation. However, its high computational complexity leads to low detection efficiency. In order to achieve fast localization and precise segmentation of defects, A two-stage inspection architecture, combining YOLOv5 and U-Net, was applied to achieve efficient real-time detection of surface defects on fish [28]. The two-stage detection model architecture and workflow are shown in Figure 5 and Figure 6, respectively. The YOLOv5 model first performs rapid coarse localization of potential defect areas, effectively eliminating redundant background computations. Subsequently, the defective region is fed into an enhanced U-Net architecture for fine-grained pixel-wise segmentation.

#### 2.5.2. Original YOLOv5 Network Structure

In Two-stage defect detection model, YOLOv5 was used to realize the fast localization of salmon surface defects, which, as a typical representative of single-stage target detection algorithms, can meet the demand for real-time production in industrialized production by virtue of the advantages of small amount of computation and fast detection speed [29]. Figure 5a–d illustrates the base network of YOLOv5, comprising four modules: Input (Figure 5a), Backbone (Figure 5b), Neck (Figure 5c), and Output (Figure 5d) [30]. The Mosaic data enhancement technique is used on the input side to enhance the diversity of samples by randomly splicing four different fish images to improve the model’s adaptability to changes in fish posing posture. The backbone network is based on the CSPDarknet structure, which slices the input image through the Focus module and then convolves it, cooperates with the C3 module to gradually extract the fish body features, and finally compresses the multi-scale features through the SPPF structure, which enhances the ability to capture the global features of defects. The neck network consists of a feature pyramid network (FPN) and a path aggregation network (PAN), where the FPN passes top-down high-level semantic features and the PAN complements bottom-up bottom-level localization features to improve the recognition of defects of different sizes through multi-scale feature fusion. The output provides 3 scales of inspection results for the localization of small, medium and large size defects [31].

#### 2.5.3. Introduction of Small Target Detection Layer

In order to solve the problem of detecting tiny defects on the surface of salmon, a new P2 small target detection layer is added to the YOLOv5 base network (Figure 5d). Technically, the P2 layer is incorporated into the Feature Pyramid Network (FPN) to leverage high-resolution, early-stage features from the backbone. Through this integration, finer spatial details are provided to the FPN, enabling minute defects to be effectively detected, which would otherwise be indistinguishable in the coarser, deeper feature maps. The detection accuracy of tiny targets can be improved by enhancing the extraction and fusion of high-resolution features. For YOLOv5 network, the input images are resized from their preprocessed dimensions of 512 × 512 pixels to meet the network requirements (640 × 640 pixels). For the input receives 640 × 640 × 3 fish body images, and four images with different poses are randomly spliced by Mosaic data enhancement technique to enrich the sample diversity and enhance the model’s ability to adapt to changes in defect location and scale. Only two downsampling operations are performed in the backbone network, containing richer information about the underlying features. The two P2 layer features obtained from top-down and bottom-up in the neck network are fused with the same scale features in the backbone network through concatenation [32]. On the output side, a new 160 × 160 P2 detector head is connected in parallel with the original three detection heads (80 × 80, 40 × 40, and 20 × 20), forming a multi-scale detection system. The output features are fused from the three input features, enabling the P2 head to handle tiny targets more efficiently and accurately.

### 2.6. Evaluation Indicators

All models were trained and evaluated on a workstation with an Intel Core i7-13700K CPU, 32 GB RAM, and an NVIDIA GeForce RTX 3080 GPU (10 GB VRAM). The inference speed (fps) and computational efficiency reported below were measured in this environment. The two-stage model had a computational load of approximately 15 GFLOPs and a GPU memory footprint of around 4.2 GB during inference. In order to objectively evaluate the performance of the U-Net Part identification segmentation model, the YOLOv5 defect detection model and the two-stage defect detection model, mean Average Precision (mAP), mean Intersection over Union (mIoU), Pixel Accuracy (PA), and processing speed (frames per second, fps) were selected as the main evaluation metrics [33]. The mAP is used to measure the combined detection accuracy of the model on multi-category targets, reflecting the overall recognition ability of the model, which contains different parts and defects of salmon. The mIoU is mainly used to evaluate the degree of overlap between the predicted region and the real labeled region in the image segmentation task, which is an important indicator of the accuracy of the segmentation boundary, and the higher value represents the better match between the segmentation result and the real labeling. The processing speed (fps), i.e., frame rate, indicates the number of images that the model can process per second, and is a key metric for evaluating the computational efficiency and real-time performance of the model. In the salmon part segmentation and defect detection task, higher mAP values indicate that the model is able to accurately distinguish between different parts and reliably identify multiple defects; higher mIoU and PA values mean more accurate segmentation boundaries at the regional and pixel levels, respectively; higher mIoU values mean more accurate segmentation boundaries; higher fps value ensures that the model is able to meet the real-time processing capability requirements of industrialized production lines. Together, these three metrics provide a comprehensive and balanced evaluation of model performance in terms of recognition accuracy, segmentation accuracy and processing efficiency [34]. The calculations are shown in Equations (1)–(7).

(1) $$R=\frac{TP}{TP+FN}$$

(2) $$P=\frac{TP}{TP+FP}$$

(3) $$AP=\int_{1}^{0}\left(P\left(R\right)dR\right)\;$$

(4) $$mAP=\frac{\sum \left(AP\right)}{n}\;$$

(5) $$mIoU=\frac{1}{k+1}\sum_{i=0}^{k}\frac{TP}{FN+FP+TP}$$

(6) $$fps=\frac{N}{T}$$

(7) $$PA=\frac{\sum_{i=0}^{k}{TP}_{i}}{\sum_{i=0}^{k}\left({TP}_{i}+{FP}_{i}\right)}$$

where R, which stands for recall, represents the ratio of targets correctly predicted by U-Net and YOLOv5 models to all targets. P, which stands for Precision, represents the accuracy of the model prediction. TP represents the number of samples where the ground truth is positive and the prediction is also positive, FP is the number of samples where the ground truth is positive but the prediction is negative, and FN is the number of samples where the ground truth is negative but the prediction is positive. n represents the total number of categories in the dataset, which in part segmentation contains the belly, loin, and back of the fish, and in defect detection contains blood and fat. N represents the total number of images used to calculate the speed. T represents the processing time. For the part segmentation task, the mean Intersection over Union (mIoU) for each sample was used as the performance indicator, as it directly quantifies the alignment between the segmented and ground-truth boundaries. For the defect detection task, the Average Precision (mAP) for each sample served as the performance indicator, given its comprehensive consideration of both precision and recall, offering a holistic reflection of detection performance. To assess the statistical significance of performance differences between models, a paired *t*-test was employed. This test compares the performance metrics of the two models on each individual sample within the identical test set. The reported *p*-value of less than 0.05 was considered statistically significant, indicating that the observed differences are unlikely to be due to random chance [35].

## 3. Results

### 3.1. Image Preprocessing Effects

**Figure 7:**
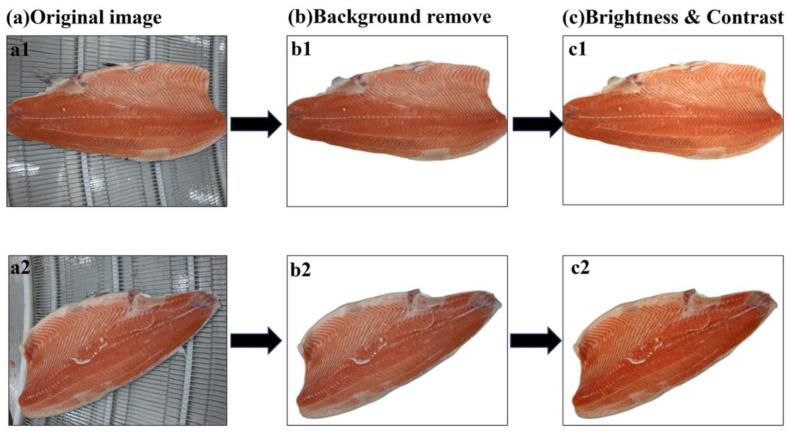
Image preprocessing effects: (**a**) Original image; (**b**) Background removee; (**c**) Brightness & Contrast. (**a1**–**c1**) and (**a2**–**c2**) represent two distinct sample processing groups, respectively.

To improve the model accuracy and generalization ability, the captured raw images are preprocessed, including background removal and adjustment of brightness and contrast. Figure 7a shows the original images with different placement positions and shooting angles. The mIoU after background removal using U-Net reaches 98.62%, indicating that the background interference is effectively excluded, and this enhancement provides high-quality input data for subsequent part segmentation and defect detection. The result of background removal based on U-Net model is shown in Figure 7b, which effectively excludes redundant information such as conveyor belts and makes the calculation of the model more targeted. Figure 7c shows the results of brightness and contrast adjustment. The adjustment of brightness and contrast makes the texture features of the image more obvious and improves the clarity of the image, so that the model can capture the feature information of the target area more accurately, thus improving the model accuracy and enabling the model to be better applied to the actual processing detection.

### 3.2. Part Identification Segmentation Model

**Figure 8:**
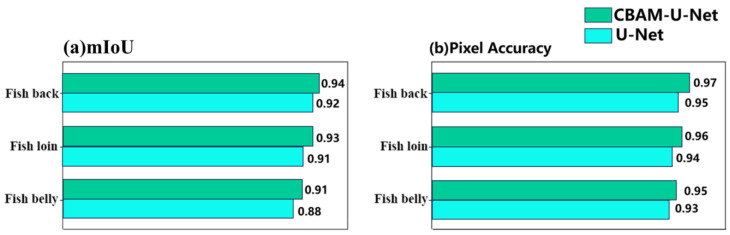
Evaluation metrics for each part: (**a**) mIoU; (**b**) Pixel Accuracy.

The part identification segmentation model was established based on an improved U-Net, which introduced a CBAM attention mechanism into the original UNET network. In order to assess the effect of improvement, an ablation experiment was performed. Under the same conditions, the original U-Net model and the U-Net model with the addition of the CBAM attention mechanism (CBAM-U-Net) are trained and tested on the site segmentation dataset, respectively, and the experimental results of the two are compared to verify the improvement effect. As shown in Table 2, the CBAM-U-Net model significantly outperforms the base U-Net model in all core performance metrics, and the mean average precision (mAP) is improved by 2.44%, indicating that the improved model has higher prediction accuracy for pixel-level segmentation of specified parts of the fish body. The mean intersection and union ratio (mIoU) improved from 93.27% to 94.33%, an improvement of 1.06%, indicating a better overlap between the model-predicted site contours and the actual results. The CBAM-U-Net model achieved a detection speed of 3.13 fps, remaining comparable to the base U-Net (2.97 fps). This result indicates that the significant improvements in segmentation accuracy were achieved without a substantial loss in processing efficiency. The advantages of the improved model can be further verified from the breakdown metrics of each part as shown in Figure 8. As can be seen in Figure 8a, in terms of the mean intersection ratio (mIoU), CBAM-U-Net outperforms the base U-Net in the key parts of the fish back, lion, and belly, in which the mIoU is, respectively, improved by 2%, 2% and 3%. As can be seen from Figure 8b, in terms of pixel accuracy, CBAM-U-Net has higher metrics than the base U-Net in all parts of the body, with a pixel accuracy of 0.95 for the belly of the fish, 0.96 for the belly of the fish, and 0.97 for the back of the fish, which further corroborates that the improved model is superior to classify individual pixels in terms of accuracy. All of the above results indicate that CBAM-U-Net is more accurate in segmenting the complex region, which is probably due to the introduction of the CBAM attentional mechanism, and the attention module strengthens the expression of key features such as fish body texture and color by adaptively adjusting the feature channel weights. Furthermore, the paired *t*-test conducted on the part segmentation task resulted in a statistically significant difference (*t*(48) = 4.82, *p* < 0.001), demonstrating the superior performance of the CBAM-U-Net model.

**Figure 9:**
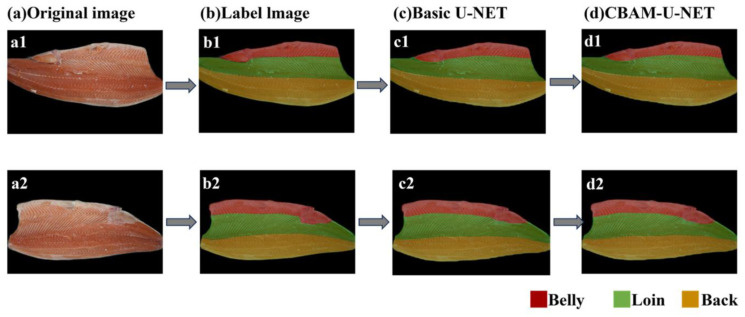
Comparison of Parts Segmentation Effect: (**a**) Original image; (**b**) Labeled image; (**c**) Basic U-Net; (**d**) CBAM U-Net. (**a1**–**d1**) and (**a2**–**d2**) demonstrate the complete processing pipeline applied to two distinct sample images.

Two pieces of fish that have been pretreated by filleting were randomly selected, and the part identification segmentation results are shown in Figure 9, where Figure 9a shows the original image, Figure 9b shows the manually labeled image, Figure 9c shows the base U-Net prediction result, and Figure 9d shows the CBAM-U-Net prediction result, where the red region is the belly of the fish, the green region is the loin of the fish, and the yellow region is the back of the fish. Comparing Figure 9c with Figure 9d, it can be seen that the predicted segmentation contours of both models fit the manually labeled desired segmentation contours in Figure 9b, with smooth overall contours, and even outperform the manually labeled contours in some details, e.g., the capture of the dividing line for the texture bumps on the surface of the fish flesh, as well as the fitting of the predicted contours to the edges of the fish body. The original U-Net model produced a small amount of feature confusion at the junction of the belly and loin of the fish, which may be due to the close proximity of the texture features of the belly and loin of the fish, leading to interference in the model’s identification of the boundary. The predicted contours of the CBAM-U-Net model are smoother compared to the original model and are more accurate in capturing texture bumps, and segmentation is better accomplished for the boundaries of the fish loin. In addition, the CBAM-U-Net model has a smaller boundary error in the transition region between the dorsal and ventral regions of the fish, which further corroborates the ability of the CBAM mechanism to accurately extract fish body features, and demonstrates that its segmentation robustness is significantly better than that of the base U-Net model in complex texture scenarios.

### 3.3. Two-Stage Defect Detection Modeling

A two-stage defect detection model combining improved YOLOv5 and U-Net was established. Comparative experiments on two-stage detection architectures using U-Net, YOLOv5, CBAM-U-Net, P2-YOLOv5, and the joint two models are conducted, respectively. As shown in Table 3, for U-NET, the mAP is only 62.50%, and the prediction speed is only 2.15 fps, which indicates that the original segmentation model has insufficient ability to recognize the tiny defects and has a large amount of redundant computations. YOLOv5 has a mAP of 80.25% and a prediction speed of 57.50 fps, indicating that compared to U-Net, YOLOv5 demonstrates significantly higher accuracy in defect detection. However, as mentioned earlier, the model is unable to output pixel-level fine contours. After introducing the CBAM and P2 into these two models, respectively, mAP improved in both cases. Although prediction speed decreased, the change was negligible. Therefore, by integrating the strengths of CBAM-U-Net and P2-YOLOv5, the two-stage defect detection model is constructed which has a highest mAP of 94.28% and a prediction speed of 7.30 fps, which demonstrates the effectiveness of the model improvement and the potential of the two-stage model in salmon defect detection. Moreover, in the defect detection task, a paired *t*-test confirmed the significant improvement of the two-stage model (*t*(61) = 9.36, *p* < 0.001), achieving a synergistic optimization of both accuracy and efficiency.

**Figure 10:**
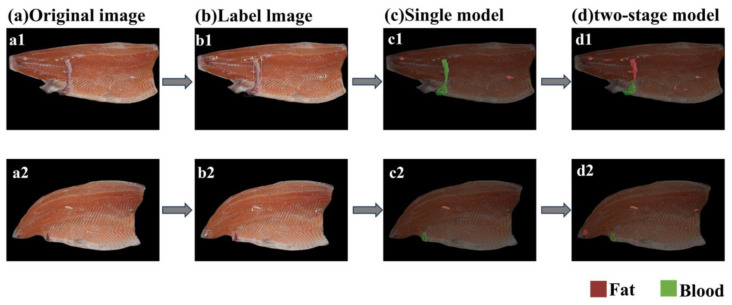
Comparison of defect detection results between the single model and the proposed two-stage model: (**a**) Original image; (**b**) Labeled image; (**c**) Result from the single model; (**d**) Result from the two-stage model. (**a1**–**d1**) and (**a2**–**d2**) demonstrate the complete detection pipeline applied to two distinct sample images.

Two pieces of fish body images with significant differences in appearance were randomly selected for comparison. The results are shown in Figure 10, where Figure 10a is the original image, Figure 10b is the manually labeled image, Figure 10c is the result of the single model detection, and Figure 10d is the result of the two-stage model detection, where the red area is fat and the green area is blood. Comparison of Figure 10c,d reveals that the overall prediction of a single model is worse. As can be seen from Figure 10(c1,d1), the former omits part of the fat region and mistakes part of the fat region for the bruise region, which may be due to the poor recognition ability of the model for tiny targets due to the random distribution of the defective targets and small area, and spends a lot of redundancy to compute the irrelevant regions in the image where the defective distributions do not exist, resulting in the poor performance of both the accuracy and efficiency. The results obtained from the two-stage model not only allow for accurate localization of the defect area, but also for more precise detection of the detailed defect contour, without mixing the fat area with the bruised area. Taken together, the prediction effect of the two-stage model is better than that of the single model, both in terms of prediction accuracy and detection efficiency, which strengthens the prediction accuracy and generalization ability of the model, and can meet the needs of the refined processing of salmon.

## 4. Discussion

The methodology developed in this study, which integrates a CBAM-augmented U-Net within a two-stage detection framework, was specifically designed and validated for salmon processing. Its core design, particularly the competence in capturing variable textures and small targets, delivers a valuable technical reference for precision fish processing and establishes a solid foundation for subsequent research. It should be noted that parasites and cuts are typically removed during manual trimming, while melanin spots are generally detectable through conventional image analysis. This is precisely why this study specifically focused on identifying fat and bruising defects. However, to further advance intelligent processing across the entire manufacturing chain, future work will integrate defect detection for melanin spots, parasites, and cutting injuries into a unified detection model. This effort to expand the model’s coverage will enhance its generalization capabilities and compensate for the shortcomings of traditional or manual inspection methods. Extending the framework to other species will require constructing corresponding datasets and performing thorough evaluation. Future research will also pursue more representative datasets, potentially leveraging Generative Adversarial Networks to synthesize rare defect samples for improved model coverage and robustness. In parallel, to boost deployment feasibility, we will explore efficient lightweight backbones such as MobileNetV3 and EfficientNet, complemented by attention mechanisms and model compression techniques including pruning and quantization. The long-term goal remains the development of a multi-task architecture capable of simultaneous portion segmentation, defect detection, and quality grading, which is essential for fully automated intelligent seafood processing.

## 5. Conclusions

This study presents a computer vision-based deep learning framework for precise salmon part segmentation and automated defect identification. The proposed CBAM-enhanced U-Net architecture achieved exceptional performance in part idetification and segmentation, attaining 96.87% mAP and 94.33% mIoU, demonstrating superior capability in distinguishing subtle tissue boundaries. For defect detection, the two-stage model synergized the rapid localization capability of P2-YOLOv5 (84.12% mAP at 58.82 fps) with the pixel-level precision of CBAM-U-Net, resulting in 94.28% mAP at 7.30 fps. The experimental results validate that the proposed method effectively addresses the challenges of fine segmentation and real-time defect detection in salmon refinement processing, achieving an optimal equilibrium between salmon refinement processing and real-time operational demands. Future work will focus on optimizing computational efficiency and extending the framework to other seafood processing applications.

## Figures and Tables

**Table 1:** Dataset composition and splitting strategy for different tasks.

Dataset Purpose	Model(s) Used	Total Images	Training Set	Validation Set	Test Set
Background Removal	U-Net	1529	1223	153	153
Part Segmentation	CBAM-U-Net	489	391	49	49
Defect Detection	YOLOv5, P2-YOLOv5	424	339	43	42
Defect Segmentation	Two-stage Model	616	493	61	62

**Table 2:** Comparison of performance metrics between U-NET and its improved model.

Model	mAP/%	mIoU/%	fps
U-NET	94.43	93.27	2.97
CBAM-U-NET	96.87	94.33	3.13

**Table 3:** Comparison of defect detection performance of different models.

Model	mAP/%	fps
U-Net	62.50	2.15
YOLOv5	80.25	57.50
CBAM-U-Net	66.26	2.17
P2-YOLOv5	84.12	58.82
Two-stage model	94.28	7.30

## Data Availability

The original contributions presented in this study are included in the article. Further inquiries can be directed to the corresponding authors.
